# Using Landscape Analysis to Test Hypotheses about Drivers of Tick Abundance and Infection Prevalence with *Borrelia burgdorferi*

**DOI:** 10.3390/ijerph15040737

**Published:** 2018-04-12

**Authors:** A. Michelle Ferrell, R. Jory Brinkerhoff

**Affiliations:** 1 Department of Biology, University of Richmond, 28 Westhampton Way, Richmond, VA 23173, USA; michelle.ferrell@richmond.edu; 2 College of Life Sciences, University of KwaZulu-Natal, 3209 Pietermaritzburg, South Africa

**Keywords:** GIS, Fragstats, Lyme disease, vector ecology, disease emergence

## Abstract

Patterns of vector-borne disease risk are changing globally in space and time and elevated disease risk of vector-borne infection can be driven by anthropogenic modification of the environment. Incidence of Lyme disease, caused by the bacterium *Borrelia burgdorferi* sensu stricto, has risen in a number of locations in North America and this increase may be driven by spatially or numerically expanding populations of the primary tick vector, *Ixodes scapularis*. We used a model selection approach to identify habitat fragmentation and land-use/land cover variables to test the hypothesis that the amount and configuration of forest cover at spatial scales relevant to deer, the primary hosts of adult ticks, would be the predominant determinants of tick abundance. We expected that land cover heterogeneity and amount of forest edge, a habitat thought to facilitate deer foraging and survival, would be the strongest driver of tick density and that larger spatial scales (5–10 km) would be more important than smaller scales (1 km). We generated metrics of deciduous and mixed forest fragmentation using Fragstats 4.4 implemented in ArcMap 10.3 and found, after adjusting for multicollinearity, that total forest edge within a 5 km buffer had a significant negative effect on tick density and that the proportion of forested land cover within a 10 km buffer was positively associated with density of *I. scapularis* nymphs. None of the 1 km fragmentation metrics were found to significantly improve the fit of the model. Elevation, previously associated with increased density of *I. scapularis* nymphs in Virginia, while significantly predictive in univariate analysis, was not an important driver of nymph density relative to fragmentation metrics. Our results suggest that amount of forest cover (i.e., lack of fragmentation) is the most important driver of *I. scapularis* density in our study system.

## 1. Introduction

The risk associated with vector borne zoonoses is changing globally in space and time due to natural and anthropogenic modifications to the environment [1,2,3]. Areas previously uninhabited by vectors due to environmental constraints or insufficient host availability can become endemic foci following major environmental or anthropogenic change, leading to the emergence or reemergence of vector-borne diseases [4,5]. Although climate constrains vector distributions on very broad scales [6,7,8], local and regional environmental determinants can have dramatic vector occurrence and behavior [9,10,11,12,13,14]. Vegetation, landscape composition, and land-use patterns can affect local climatic conditions and affect vector habitat suitability [15,16,17] and landscape and land-use affect host abundance in ways that can impact vector–host interactions and pathogen transmission dynamics [10,18,19,20]. For these reasons, the geographic distribution of a vector may not coincide with spatial heterogeneity in risk to diseases associated with that vector [21]. Ticks are unique among hematophagous arthropods in that they rely on vertebrate hosts for prolonged feeding bouts and can thus be dispersed over large distances in a relatively short period of time [22,23]. Therefore, occurrence of ticks in a given location is a function of local environmental conditions being conducive to survival and contact with suitable hosts, as well as processes that allow ticks to colonize a given habitat or site, such as patterns of habitat use by various host species. These processes occur at different scales; for example, climate suitability analysis may suggest that a particular site is supportive of a vector population yet availability of hosts and other species or mechanisms of dispersal to that site may limit vector occurrence [24,25,26]. For this reason, it is important to identify patterns and drivers of vector-borne disease risk that may operate at different scales [6,9,10,11,13,14].

The black-legged tick, *Ixodes scapularis*, serves as the primary vector for several zoonotic infections, including Lyme disease (caused by the bacterium *Borrelia burgdorferi* sensu stricto,) which is the most prevalent vector-borne disease in the United States [27]. Areas with high levels of Lyme disease incidence have been positively correlated with high population densities of host seeking *I. scapularis* ticks [28] but there are also areas where these ticks occur but *I. scapularis*-borne infections are uncommon [21,29,30]. Potential reasons for the imperfect relationship between vector occurrence and disease risk include: (1) variation in vector behavior in ways that impacts vector-enzootic host (or human) interaction, and (2) variation in relative abundance of hosts that have different reservoir competence [9,10,18,19]. Under the dilution effect hypotheses (sensu [31]), forest fragmentation or other land-use change that increases the relative abundance of high-competence reservoir species without affecting overall numbers of vectors can increase production of infected vectors [32], and thus disease risk. In the case of the Lyme disease system, where links between landscape configuration, host biodiversity, and disease risk were first elucidated, it has been suggested that landscape changes such as forest fragmentation, result in increased relative abundance of the highly reservoir-competent white-footed deermouse, *Peromyscus leucopus*. Elevated abundance of this host, in turn, may result higher infection prevalence with *B. burgdorferi* in *I. scapularis* ticks that are feeding proportionally more on this host [31,33]. While there is theoretical and empirical evidence for a positive relationship between forest fragmentation and various aspects of enzootic *B. burgdorferi* transmission and Lyme disease risk, this is not a universal phenomenon and may be confounded by variation in human behavior and other factors (see [34] for a recent review of this topic).

Lack of a consistent relationship between habitat fragmentation and increased entomological risk for Lyme disease may be explained by impacts of effects of overall land cover context and/or change on white-tailed deer (*Odocoileus virginianus*) populations. *O. virginianus* is the principal host for adult *I. scapularis* but is not a competent reservoir for *B. burgdorferi*. Thus, landscape change that increases deer abundance, irrespective of impacts on *P. leucopus*, could result in increased *I. scapularis* density (i.e., more opportunities for tick reproduction). However, addition of more immature *I. scapularis* could reduce infection prevalence with *B. burgdorferi* if deer are redirecting potentially-infective bloodmeals for immature *I. scapularis* from *P. leucopus*. Local reduction or elimination of deer populations has been shown to reduce host-seeking *I. scapularis* abundance [35,36,37] but links between relative abundance of deer and *I. scapularis* infection prevalence with *B. burgdorferi* are less clear [38,39,40]. Tick host-seeking behavior may also be dependent on landscape context wherein vegetation physiognomy and microclimate determine where, when, and for how long ticks are able to search for hosts [10,15,17]. Timing of host-seeking can determine which host types a tick is likely to encounter and can thus affect population infection prevalence when different hosts vary in reservoir competence [41]. The scale at which landscape context impacts host occurrence and behavior may be very different from the scale at which ticks are impacted. Moreover, different hosts (i.e., mice and deer) may respond to landscape configuration changes at different scales and in ways [24,42,43,44] that are important to pathogen transmission processes and ultimately disease risk.

In Virginia, Lyme disease incidence has increased over 300% since 2006 with significantly broadened geographic distribution over the past decade [30,45,46]. Interestingly, areas of highest increases in incidence in Virginia are inconsistent with environmental models of predicted *I. scapularis* habitat suitability. Spatial models of *I. scapularis* abundance [47] and infection prevalence with *B. burgdorferi* [29] identified low-elevation and relatively humid coastal areas to be associated with the highest host-seeking tick densities and Lyme disease risk whereas it is higher-elevation inland localities have been observed to have the largest increases in incidence [45,46] and highest numbers of host-seeking *I. scapularis* [30]. Molecular data suggest that growing and spatially expanding populations of *I. scapularis* may account, in part, for increasing disease risk [48], and analysis of human case data revealed interspersion of forested and herbaceous (i.e., scrubland, grassland, and agricultural) land cover to be a significant predictor of Lyme disease [49]. The rapid rise in Lyme disease in Virginia in areas previously predicted to be low-risk demands further investigations into potential impacts of landscape context and environmental change on abundance of *I. scapularis* and enzootic maintenance of *B. burgdorferi*. Furthermore, aspects of anthropogenic environmental change could impact other human-biting and pathogen-transmitting ticks. For this reason, we compared landscape drivers of *I. scapularis* at various with against predictors of abundance for the lone star tick, *Amblyomma americanum*, which is also dependent on deer to complete its lifecycle as well as the American dog tick, *Dermacentor variabilis*. Immature *D. variabilis* parasitize many of the same host species as *I. scapularis*, but different hosts (medium-sized mammals) as adults. Although distributions of these tick species overlap in Virginia, each has different tolerance for temperature and relative humidity and are found in different abundance in different habitats ([50], Brinkerhoff unpublished data).

Our goal for this study was to identify land cover characteristics associated with high tick abundance and infection prevalence with *B. burgdorferi* to test make inferences about processes that drive spatial variation in human risk to tick-borne infections. *I. scapularis* occurs throughout our study system, albeit at variable abundance [30,48], and we thus assume that all sites are capable of supporting the physiological requirements of this species. To these ends, we made no effort to account for heterogeneity in microclimate, even though this has been showing to impact tick host-seeking behavior (e.g., [10,17]). Rather, our goal was to explore ways larger-scale landscape context might affect *I. scapularis* abundance through impacts on host habitat use. Because *I. scapularis* populations are dependent on deer, we hypothesized that the amount and configuration of forest cover at scales relevant to deer movement (i.e., tens of kilometers [42,43]) would be the primary determinant of tick abundance for *I. scapularis.* We further predicted that infection of immature *I. scapularis* ticks with *B. burgdorferi* would be most strongly impacted by local-scale landscape configuration characteristics that affect abundance and movement of *P. leucopus* (<1 km [44]). Similarities and differences in predictor variables and scale-dependence among these three human-biting ticks that overlap to some extent in host utilization may shed light on tick ecology and drivers of tick–host interactions.

## 2. Materials and Methods

### 2.1. Study Sites and Tick Collection

**Figure 1:**
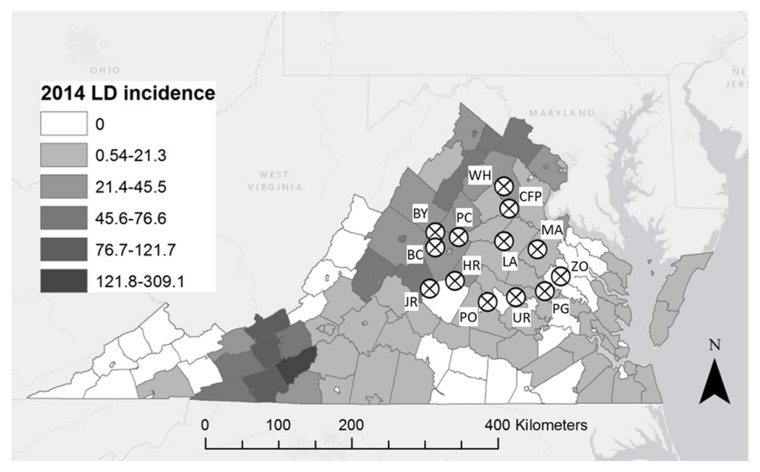
Map of study sites (circles) throughout central Virginia sampled for *I. scapularis*, *A. americanum*, and *D. variabilis* in May and June 2014. Counties are shaded based on 2014 Lyme disease incidence value. BC: Beaver Creek Park; BY: Patricia Ann Byrom Forest Reserve Park; UR: Graveyard; HR: Hardware River WMA; JR: James River WMA; LA: Lake Anna State Park; MA: Mattaponi; CFP: CF Phelps WMA; PG: Pole Green Park; PO: Powhatan WMA; PC: Preddy Creek; WH: Whitney; ZO: Zoar State Forest.

We conducted field sampling for ticks at thirteen sites in central Virginia in counties that range widely in human Lyme disease incidence (Figure 1) and where previous studies reported significant variation in *I. scapularis* density and infection prevalence with *B. burgdorferi* [30]. From May to July 2014 we sampled each site for ticks by drag sampling with a 1 × 1 m corduroy cloth along both sides of five haphazardly selected 100 m transects, for a total 1000 m^2^ of sampling area per site. Visits to sites were separated by at least two weeks with an average of 29 days between visits. To standardize tick collection among sites and to minimize heterogeneity resulting from edge effects, all sampling was conducted in interior (i.e., >100 m from forest edge) deciduous or mixed-deciduous forest and we selected transects with minimal understory vegetation to maximize drag cloth contact with leaf litter on the ground. During each sampling occasion, we stopped to remove ticks every 20 m and placed them into vials containing 70% ethanol. Ticks were identified to species by light microscopy using dichotomous keys [50], and the density of *I. scapularis* and *A. americanum* ticks was determined as the average number of ticks for that species per transect at each site. We calculated infection prevalence with *B. burgdorferi* as the number of infected ticks divided by the total number of *I. scapularis* ticks sampled at that site and calculated density of ticks as the number of individuals of a given species per 200 m^2^ averaged across all five transects and two sampling occasions.

### 2.2. Molecular Detection of B. burgdorferi

All collected *I. scapularis* ticks were dried, flash frozen in liquid nitrogen, and pulverized using sterilized pestles in individual micro-centrifuge tubes. We extracted DNA using a Qiagen DNeasy Blood and Tissue Kit (QIAGEN, Valencia, CA, USA) following the manufacturer’s protocols with at least a 4 h incubation at 56 °C to allow for tissue degradation and cell lysis. Presence of *B. burgdorferi* DNA was determined by nested PCR detection of the outer surface protein c (*osp*C) gene and a portion of the 16S–23S ribosomal intergenic spacer region [51]. Amplicons were visualized via gel electrophoresis and staining with ethidium bromide and amplicons from PCR-positive ticks were purified using a QIAquick PCR Purification Kit (QIAGEN, Valencia, CA, USA) for DNA sequencing.

### 2.3. Spatial Analyses

**Figure 2:**
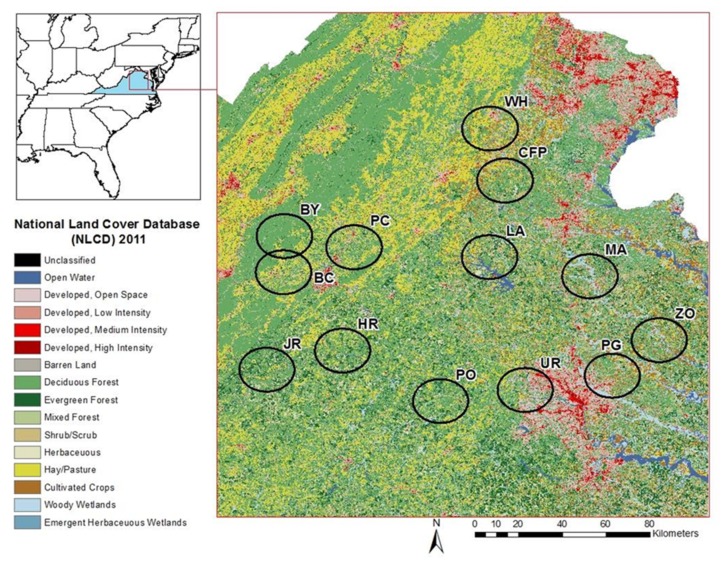
Sampling sites surrounded by 10 km diameter buffers (black circles) and superimposed on 2011 NLCD imagery with condensed land cover categories indicated at left. BC: Beaver Creek Park; BY: Patricia Ann Byrom Forest Reserve Park; UR: Graveyard; HR: Hardware River WMA; JR: James River WMA; LA: Lake Anna State Park; MA: Mattaponi; CFP: CF Phelps WMA; PG: Pole Green Park; PO: Powhatan WMA; PC: Preddy Creek; WH: Whitney; ZO: Zoar State Forest.

To collect land cover data at an individual site we averaged the latitude and longitude for the beginning of each transect at that site and extracted land cover data from the 2011 National Land Cover Database (NCLD) at 30 m resolution [52] using 10, 5, and 1 km buffers around the site centroid (Figure 2). We selected these buffers because the larger radii correspond average daily movement (approx. 3–7 km; [42]) and average juvenile dispersal (7–8 km; [43]) distances for *O. virginianus* and the smaller radius (1 km) encompasses dispersal distance and inter-day movement rate estimates for *P. leucopus* [44]. We projected all of the data sets to Albers USA contiguous equal area conic (USGS version) to give all of the pixels equal area. We reclassified the mixed forest to have the same value as deciduous forest using ArcGIS 10.2.2 (Esri, Redlands, CA, USA) to take into account the combined effect of mixed and deciduous forest on *I. scapularis* tick density; this reclassification was done because none of our forest sampling sites are old growth forest and most are a mix of pine and hardwood species.

We used the reclassified NCLD extracted GIS buffers (1 km, 5 km, and 10 km) in Fragstats 4.4 [53] to calculate landscape and fragmentation metrics at the class and landscape level. At the class level, metrics of land cover are compared between all patches of a given class (i.e., deciduous forest), and at the landscape level, the metrics take into account the whole landscape. Guided by previous landscape analysis of *I. scapularis* abundance and infection prevalence with *B. burgdorferi* [29], we focused on the combined effects of mixed and deciduous forest, and analyzed each of the following landscape metrics that are indicative of fragmentation and forest cover: percent land cover (PLAND), number of patches (NP), largest patch index (LPI), mean patch size (MPS), total edge (TE), Shannon’s diversity index (SHDI), Simpson’s diversity index (SIDI), and the Euclidean nearest neighbor distribution (ENN; calculations and descriptions in Table 1).

### 2.4. Statistical Analysis

All predictor variables were z-transformed to ensure equivalence of coefficients in regression models. In addition, the elevation parameter was natural log transformed for normalization prior to analysis. We eliminated the Euclidean nearest neighbor mean and coefficient of variation variables for the 1 km buffer because it was undefined for some sites; in short, there were some sites that had no discrete patches of forest within 1 km because the entire area within the buffer was classified as forest. We ran separate analyses using estimated *I. scapularis* density and *I. scapularis* infection prevalence with *B. burgdorferi* as response variables. For the abundance analysis, the response variable was square-root transformed to ensure normality of distribution and residuals and for predictor variables within each landscape buffer class (1, 5, or 10 km) we assessed collinearity and removed offending variables. We then used combined forward and backward stepwise selection (corrected Akaike information criterion (AICc) used as selection criterion and exploring up to 10 steps), implemented with the *glmselect* procedure in SAS 9.4 (SAS Institute, Inc., Cary, NC, USA) to identify the best predictor variables within a landscape buffer class; in cases where multiple models with different numbers of variables were included in the set of models with ΔAICc ≤ 2, we opted to include the largest number of predictor variables in the next step of analysis. To assess relative importance of the best-fitting variables from each buffer class, we used a comprehensive model selection approach implemented with ‘proc reg’ in SAS 9.4 wherein the top five models for each number of predictor variables (1 to *n*, depending on how many were identified in the previous step) were reported and sorted by AICc. In addition to the variables chosen from each buffer class, we included elevation as a potential predictor given its emergence as a potential determinant of *I. scapularis* density in prior studies (e.g., [47]). We assessed across-scale collinearity within a model set by using the condition index and used the variance inflation factor (VIF) and decomposed proportion of variance for individual variables to diagnose which variables contributed most to problems of collinearity [54]. We removed offending variables from the model until the condition index for a set of variables was less than 10 and variable-specific VIF values were <10. We characterized the relative importance of each predictor variables by identifying the number of times each variable was included in models with ΔAICc ≤ 2. In addition to developing models to explore *I. scapularis* abundance, we used the same process to test for landscape drivers of *A. americanum* and *D. variabilis* occurrence.

To model *I. scapularis* infection prevalence with *B. burgdorferi*, we used the same conceptual approach but this time we used a Poisson rate model with ‘proc hpgenselect’ in SAS 9.4. We also weighted (offset) the infection prevalence estimate by the natural log of the total number of ticks collected at each site because precision of infection prevalence estimates is strongly affected by sample size. We used stepwise selection with liberal entry and remaining *p*-value thresholds (0.4 and 0.2, respectively) to explore parameter space and identify the best explanatory variables within and among buffer classes. Candidate models in were again evaluated based on ΔAICc.

## 3. Results

We collected 2458 ticks of three species: 2090 *A. americanum* (1 larva, 1953 nymphs, 136 adults), 299 *I. scapularis* (296 nymphs, 3 adults), and 69 *D. variabilis* (all adults, Table 2). The population density for *I. scapularis* ranged from 0.1 to 8.6 ticks per 200 m^2^. Infection with *B. burgdorferi* was found at 6 sites out of 13 and infection prevalence with *B. burgdorferi* ranged from 0.0 to 0.33 (Table 2).

Following screening of land-use variables at each buffer class, the regression model used to predict *I. scapularis* density included the following variables: total forest area (1 km), total linear forest edge (5 km), Shannon’s diversity index (5 km), average Euclidean nearest neighbor (10 km), average forest patch size (10 km), Shannon’s diversity index (10 km), and elevation. Assessment of multicollinearity led to the removal of Shannon’s diversity index (5 km) and average forest patch size (10 km) variables and reduced the condition index of the final model from >31 to 6.5. We identified two models within ΔAICc ≤ 2 (Table 3); both of the top models had three predictor variables (plus intercept) and two of these three variables, total edge at 5 km and Shannon’s diversity index at 10 km, we common to both models. Interestingly, both of these variables, as well as elevation, were associated with negative parameter estimates; increasing inter-patch distance within the 10 km buffer was the only parameter that had a positive coefficient. Within-buffer selection identified the following variables as potential predictors of *I. scapularis* infection prevalence with *B. burgdorferi*: number of patches at all buffers, average patch area at all buffers, variation in nearest patch distance at 5 km, Shannon’s diversity index at 5 and 10 km, and proportion of forest cover at 10 km. Due to substantial collinearity (r > 0.8) with variables in other buffer classes, all 5 km variables except for variation in nearest forest patch distance were removed, as was proportion of forest cover at 10 km. Model selection resulted in a final equation with two variables: variation in nearest forest patch distance (coefficient = −172.2, *p* = 0.001) and elevation (coefficient = 140.7, *p* = 0.001) (Table 4). All variables available for selection were significant predictors of infection prevalence in univariate analysis.

Five landscape variables—average patch area within all buffers, Shannon’s diversity index at 10 km, and elevation—were selected among for the final *D. varibilis* density model analysis and average patch area at 10 km was removed being identified as having negative impact on collinearity metrics (VIF for this variable >200). Final assessment of the remaining five variables (four landscape variables as well as elevation) identified one models with a single variable with the lowest AICc (Table 5) and no other models within ΔAICc ≤ 2. Average forest patch area at 5 km was the only predictor variable in the top model and was associated with a positive coefficient; the variables included in other models (average forest patch area at 1 km, Shannon’s diversity index at 10 km, and elevation) all had small average coefficients with relatively large standard error (Table 5). Analysis of *A. americanum* abundance identified no significant predictor variables at any buffer size at alpha = 0.05. Likewise, there was no significant effect of elevation on *A. americanum* abundance (F = 0.07, *p* = 0.8, adjusted r^2^ = −0.08).

## 4. Discussion

In this study we demonstrated that environmental variables at different spatial scales can be used to predict *Ixodes scapularis* and *Dermacentor variabilis*, but not *Amblyomma americanum*, density. Although we found different sets of predictor variables for each tick species and for *I. scapularis* infection prevalence with *B. burgdorferi*, we did not find strong support for our hypothesis that *I. scapularis* infection prevalence would be driven by local (i.e., within 1 km) conditions while *I. scapularis* abundance would be driven by larger-scale landscape variation. *I. scapularis* abundance was generally associated with larger-scale predictor variables but variables across scales were associated with *B. burgdorferi* infection prevalence (Table 3 and Table 4). It is noteworthy that previously-identified factors associated with increased *I. scapularis* abundance and elevated Lyme disease risk such as greater amounts of forest edge and high interspersion of forest with other habitats [49,55], were found to be negatively associated with *I. scapularis* abundance in this study (Table 3). That the relative abundance of *I. scapularis* is generally consistent from year to year (Brinkerhoff and Ferrell unpublished data, Figure S1) suggests that there are site-level factors that determine habitat suitability and/or population persistence for this species and our analyses suggest that there are larger-scale aspects of landscape context that are important predictors of *I. scapularis* density, and potentially *B. burgdorferi* infection prevalence. Whether the impacts of landscape configuration are important because of vertebrate habitat use and behavior, microclimate variation, or some other factor is beyond the scope of this study but is worthy of future investigation. We have previously shown that Lyme disease incidence in Virginia is associated with the density of infected *I. scapularis* ticks [30] and that counties in Virginia that have experienced the largest increase in incidence also tend to spatially and/or numerically expanding *I. scapularis* populations [48]. Spatial analysis of vector-borne disease risk is increasingly recognized as an effective tool for tracking and predicting outbreaks but there are important limitations to these approaches (see [56] for a recent review). Linking statistical predictors of vector occurrence and vector-borne disease risk with mechanistic explanations is challenging but will allow more precise forecasting of changing patterns of pathogen transmission in the face of anthropogenic environmental change.

Interestingly, we were able to find significant environmental determinants of *D. variabilis* density, namely positive effects of forest patch area within 5 km of our sampling site centroid, but not *A. americanum* density, suggesting that local and regional landscape structure may affect some ixodid tick species more than others. Although both of these species are widespread, stronger association with specific habitats in *D. variabilis* compared to *A. americanum* is consistent with previous statewide records [50]. The difference in suites of predictor variables for different metrics are likely reflective of the different environmental processes that drive tick population dynamics as well as interactions with pathogen reservoirs. Immature *D. variabilis* and *I. scapularis* occur on some of the same hosts and in the same habitats, but immature *D. variabilis* were not encountered during tick collection, suggesting that their host-seeking behavior does not make them well-suited to drag sampling. Thus, our estimates of *D. variabilis* density and subsequent statistical modeling efforts to identify drivers of its occurrence are likely compromised by non-representative sampling. Adult *D. variabilis* are easily collected during drag sampling but tend to occur in specific microhabitats and microclimates and are more abundant outside of closed-canopy deciduous forest [Brinkerhoff unpublished data] than in the forest interiors where we concentrated our sampling. *A. americanum* immatures and adults are readily collected in a variety of habitats in Virginia and this was by far the most commonly encountered tick species in this study. Given the reliance of this species on white-tailed deer for bloodmeals at all stages, it is somewhat surprising that there were not strong landscape-level predictors of its density that could be related to deer abundance and/or habitat use. Our inability to develop viable statistical models to describe the abundance of this species could be due to the high abundance of *A. americanum* at nearly all sites or to its hardiness and ability to survive and thrive under variable environmental conditions [57].

Host occurrence, habitat use, and environmental tolerances drive local abundance of ticks. Given that deer are critical to completion of the *I. scapularis* life cycle, we expected to see highest tick abundance at sites dominated by habitats preferred by deer, such as forest/herbaceous edges (e.g., [58]). Positive associations between deer abundance, *I. scapularis* abundance, and forest fragmentation have been described previously (e.g., [59,60]) and forest fragmentation, coupled with smaller forest patch size, may result in increased *I. scapularis* infection prevalence with *B. burgdorferi*, likely through landscape-mediated impacts on small mammal abundance [32,49]. Our results do not provide support for a positive relationship between forest fragmentation and *I. scapularis* abundance or Lyme disease risk. In fact, sites with the highest tick abundance and infection prevalence with *B. burgdorferi* were characterized by low total forest edge and high amounts of intact forest at large spatial scales (Table 3 and Table 4). While this finding contrasts with results from other geographical regions [32,61], it is also inconsistent with analyses of human case data in Virginia showing that high amounts of forest-herbaceous edge are predictive of Lyme disease incidence [49]. However, these inconsistencies are not necessarily problematic and are consistent with recent research that failed to show links between pathogen infection prevalence and forest fragmentation [62]. Similarly, Brownstein et al. [61] noted that incidence data may be decoupled from estimated entomological risk and case data aggregated by census block (e.g., [55]) may not correspond to site of exposure. These inconsistencies may arise because the link between increased edge habitat and higher small mammal abundance is not universal: Heske [63] found no consistent differences in small or medium-sized mammal abundances in forest interior versus forest-field edge. Moreover, deer habitat use and behavior are driven by energy budgets and perceived predation risk in addition to availability of forage [64] and thus relationships between deer abundance and forest edge may not be universal. It is likely, therefore, that the relationship between landscape configuration and densities of mammalian species, and especially tick-borne pathogen cycling, are highly context- and system-dependent. Taken as a whole, our results demonstrate that abundances of two of the tick species in our study can be predicted with a relatively small number of landscape-level predictor variables. However, we were not able support our hypotheses about how the scales at which different predictors operate are reflective of patterns of host movement and habitat use, suggesting that either other mechanisms account for the association between landscape context and tick occurrence, or that these landscape metrics are not strong predictors of host habitat use.

## 5. Conclusions

In our study system, across a gradient of Lyme disease incidence and among sites with highly variable landscape context, we found very strong drivers of abundance for two of three human-biting tick species. This outcome is promising for more predictive efforts to identify disease clusters and emerging endemic foci for vector-borne diseases. However, we also note that tick-borne disease systems are highly dynamic and identification of a suite of predictor variables from one study may or may not be useful or relevant as vector populations grow and move. For example, risk maps parameterized based on spatially-extensive sampling efforts (e.g., [28,29,47]) may fail to identify local drivers of pathogen transmission in emerging hotspots [46] just as local vector control efforts may or may not have impacts beyond application sites [25,35,65]. Suites of significant predictor variables change among study sites and study periods [55] suggesting that local processes and/or environmental conditions can have larger effects on vector occurrence than variables collected at other scales [5,9,10,12,14,16,17,18,24,56,66,67]. We also note that spatial analysis of human case data may yield different outcomes than analysis of vector occurrence (e.g., [30,45,49]). These discrepancies can arise if residential address is different from site of pathogen exposure [68] or if different processes lead to human-vector encounter rates and vector population dynamics in natural systems [61]. We note that we can do little more than speculate about mechanisms linking landscape context with tick density and infection prevalence because we were not able to collect data on mammal abundance or behavior. Furthermore, we have no data about relative survival or host-seeking behavior of *I. scapularis* or other tick species at our study sites. Still, this is the first study of landscape determinants of *I. scapularis* abundance in an area where Lyme disease incidence has increased dramatically over the course of just a few years and it is among a small number studies explicitly addressing hypotheses about mechanisms of tick abundance and infection prevalence that might be linked with landscape-level processes. As Lyme disease continues to expand geographically throughout Virginia and other locations, it will be important to further develop and refine our understanding of enzootic pathogen transmission dynamics and tick abundance.

## Figures and Tables

**Table 1:** Description of landscape metrics, modified from [43].

Landscape Metrics	Description
Percent land cover (PLAND)	Proportion of forest pixels within a buffer
Number of patches (NP)	Total number forest patches
Largest patch index (LPI)	Area of the largest forest patch, expressed as a percentage of total landscape area
Mean patch size (MPS)	Average forest patch size (ha)
Total edge (TE)	Sum of length of all edge segments for forest patches
Shannon’s diversity index (SHDI)	Negative sum, across all land cover types, of the proportional abundance of each land cover type multiplied by that proportion
Simpson diversity index (SIDI)	One minus the sum, across all land cover types, of the proportional abundance of each land cover type squared
Euclidean nearest neighbor distance distribution (ENN)	Shortest straight-line distance (m) to the nearest neighboring forest patch

**Table 2:** Tick densities per 200 m^2^ for each field site. Infection prevalence indicates proportion of ticks that tested PCR-positive for *Borrelia burgdorferi.* Two-letter site codes are indicated by each site name and WMA stands for wildlife management area. Density estimates are per 200 m^2^ and are averaged among five transects and two site visits.

		*Amblyomma Americanum*	*Dermacentor Variabilis*	*Ixodes Scapularis*	
Sites	Elevation (m)	Density	Density	Density	Infection Prevalence
Beaver Creek Park (BC)	177.1	3.6	0	1.8	0.06
Byrom (BY)	366.4	18.4	4.2	8.6	0.15
Graveyard (UR)	82.6	18.8	0	0.9	0.33
Hardware River WMA (HR)	91.4	16.4	0.1	1.8	0
James River WMA (JR)	146.9	34	0.5	1.4	0.07
Lake Anna State Park (LA)	116.5	15.4	0.1	0.6	0
Mattaponi (MA)	30.2	12.5	0.1	1.2	0.17
CF Phelps WMA (CFP)	105.2	13.2	0.2	1.5	0
Pole Green Park (PG)	50.6	19.1	0	1	0
Powhatan WMA (PO)	127.1	21.9	0.3	2.3	0
Preddy Creek (PC)	149	10	0	1.7	0
Whitney (WH)	138.1	8.6	0.1	4.1	0.05
Zoar State Forest (ZO)	29.9	8.9	0	0.1	0

**Table 3:** Parameter estimates and AICc scores for the top eight (models with ΔAICc ≤ 2 in bold) models used to predict average *I. scapularis* density among 13 sites in Virginia. This suite of variables had a maximum condition index of 6.5 and no variables had a variance inflation factor higher than 6.5.

		Parameter Estimate				
r^2^	AICc	AREA_MN(1)	TE(5)	SHDI(10)	ENN(10)	ELEV
**0.905**	**−20.96**		**−0.31**	**−0.86**	**0.41**	
**0.903**	**−20.66**		**−0.73**	**−0.67**		**−0.38**
0.854	−18.85			0.62	−1.10	
0.914	−17.92		−0.51	0.24	−0.80	−0.20
0.911	−17.46	0.10	−0.28	0.42	−0.82	
0.873	−17.21	0.18		0.62	−0.99	
0.872	−17.05			0.65	−1.03	0.16
0.832	−17.00		−0.55		−0.54	
Mean parameter estimate (all models)		0.94	−0.46	−0.76	0.27	−0.19
Parameter st err		0.14	0.27	0.25	0.25.	0.23

**Table 4:** Results from model selection analysis to explain variation in *I. scapularis* infection prevalence with *B. burgdorferi* as a function of land-use characteristics among 13 sites in central Virginia. All variables were significant at *p* < 0.01 but the five-parameter model indicated at step four was the model associated with the lowest AICc. Variable descriptions are found in Table 1.

Step	Description	Effects	Chi-Square	Pr > ChiSq	AICc
0	Initial Model	1			19.78
1	ONE_NP entered	2	1.73 × 10^2^	<0.0001	−285.58
2	TEN_TE entered	3	1.00 × 10^11^	<0.0001	−182.79
3	TEN_NP entered	4	2.70 × 10^13^	<0.0001	−237.55
4	elevation entered	5	2.20 × 10^11^	<0.0001	−490.21
5	TEN_NP removed	4	3.39 × 10^−2^	0.8538	−459.28

**Table 5:** Parameter estimates and AIC scores for the top eight models used to predict average *D. variabilis* density among 13 sites in Virginia. This suite of variables had a maximum condition index of 5.5 and no variables had a variance inflation factor higher than 7.0.

		Parameter Estimate			
r^2^	AICc	AREA_MN(1)	AREA_MN(5)	SHDI(10)	ELEV
0.84	−20.14		0.91		
0.84	−17.87		1.04	0.15	
0.84	−17.70	−0.11	0.99		
0.84	−17.30		0.91		0.00
0.85	−14.84	−0.11	1.13	0.16	
0.84	−14.42		1.06	0.16	−0.02
0.84	−14.25	−0.11	1.01		−0.02
0.85	−10.57	−0.12	1.17	0.16	−0.04
Mean parameter estimate (all models)		−0.11	1.17	0.16	−0.04
Parameter st err		0.21	0.36	0.25	0.21

## Supplementary Materials

The following are available online at http://www.mdpi.com/1660-4601/15/4/737/s1, Figure S1: Abundance of each tick species in this studied at five sites samples in 2013 (x-axes) and 2014 (y-axes); shading represents 95% confidence interval around line of best fit. Sampling methodology was identical in both years. Abundance of *I. scapularis* in 2013 was significantly (r = 0.95, *p* = 0.01) correlated with abundance in 2014; no such relationship was found for *A. americanum* (r = 0.62, *p* = 0.27) or *D. variabilis* (r = −0.31, *p* = 0.62).
