# Supplementary material for: Using Landscape Analysis to Test Hypotheses about Drivers of Tick Abundance and Infection Prevalence with Borrelia burgdorferi
Source: Int J Environ Res Public Health. 2018 Apr 12;15(4):737. doi: 10.3390/ijerph15040737 (PMC5923779; doi:10.3390/ijerph15040737)
Supplement: Supplementary file 1 [file ijerph-15-00737-s001.pdf]

International Journal of
Environmental Research and Public Health

MDPI

Supplementary Materials: Using Landscape Analysis to Test Hypotheses about Drivers of Tick Abundance and Infection Prevalence with *Borrelia burgdorferi*

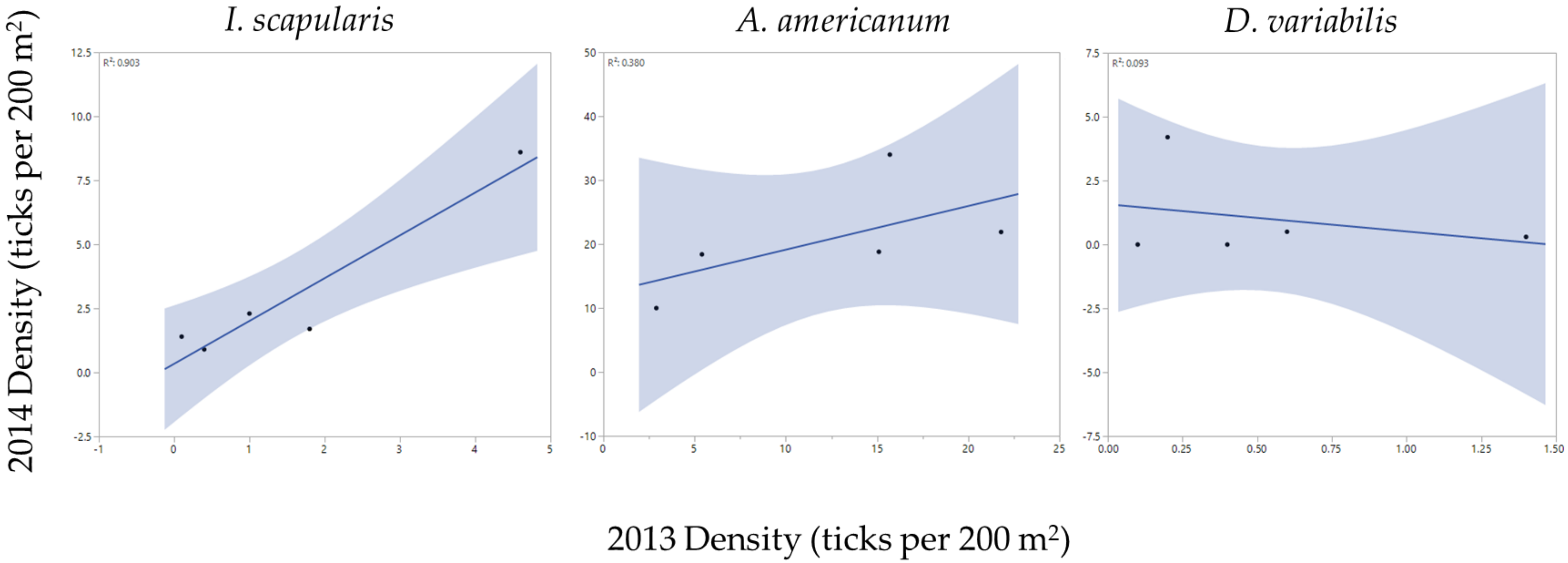

Figure S1. Abundance of each tick species in this studied at 5 sites samples in 2013 (x-axes) and 2014 (y-axes); shading represents 95% confidence interval around line of best fit. Sampling methodology was identical in both years. Abundance of *I. scapularis* in 2013 was significantly (r = 0.95, P = 0.01) correlated with abundance in 2014; no such relationship was found for *A. americanum* (r = 0.62, P = 0.27) or *D. variabilis* (r = -0.31, P = 0.62).

© 2018 by the authors. Submitted for possible open access publication under the terms and conditions of the Creative Commons Attribution (CC BY) license (http://creativecommons.org/licenses/by/4.0/).
